# Amalgamation of 3D structure and sequence information for protein–protein interaction prediction

**DOI:** 10.1038/s41598-020-75467-x

**Published:** 2020-11-05

**Authors:** Kanchan Jha, Sriparna Saha

**Affiliations:** Department of Computer Science and Engineering, Indian Institute of Technology Patna, Patna, Bihar 801103 India

**Keywords:** Computational biology and bioinformatics, Classification and taxonomy

## Abstract

Protein is the primary building block of living organisms. It interacts with other proteins and is then involved in various biological processes. Protein–protein interactions (PPIs) help in predicting and hence help in understanding the functionality of the proteins, causes and growth of diseases, and designing new drugs. However, there is a vast gap between the available protein sequences and the identification of protein–protein interactions. To bridge this gap, researchers proposed several computational methods to reveal the interactions between proteins. These methods merely depend on sequence-based information of proteins. With the advancement of technology, different types of information related to proteins are available such as 3D structure information. Nowadays, deep learning techniques are adopted successfully in various domains, including bioinformatics. So, current work focuses on the utilization of different modalities, such as 3D structures and sequence-based information of proteins, and deep learning algorithms to predict PPIs. The proposed approach is divided into several phases. We first get several illustrations of proteins using their 3D coordinates information, and three attributes, such as hydropathy index, isoelectric point, and charge of amino acids. Amino acids are the building blocks of proteins. A pre-trained ResNet50 model, a subclass of a convolutional neural network, is utilized to extract features from these representations of proteins. Autocovariance and conjoint triad are two widely used sequence-based methods to encode proteins, which are used here as another modality of protein sequences. A stacked autoencoder is utilized to get the compact form of sequence-based information. Finally, the features obtained from different modalities are concatenated in pairs and fed into the classifier to predict labels for protein pairs. We have experimented on the human PPIs dataset and *Saccharomyces cerevisiae* PPIs dataset and compared our results with the state-of-the-art deep-learning-based classifiers. The results achieved by the proposed method are superior to those obtained by the existing methods. Extensive experimentations on different datasets indicate that our approach to learning and combining features from two different modalities is useful in PPI prediction.

## Introduction

The main building block of the living organisms is the protein. It takes part in various processes of life activities. These activities include hormone regulation, metabolism, signal transduction, cell transcription, and replication^1,2^. Most of these activities involve different types of protein interactions. The study of protein–protein interactions helps in understanding the biological processes and assists in the development of new drugs^3–7^ and in exploring the growth and causes of diseases^8^. Also, the knowledge of PPIs with gene interaction network analysis is useful to predict drug targets, for example, in the case of pathogenic bacteria^9–13^. Several high-throughput experimental techniques such as yeast two-hybrid (Y2H)^14,15^, tandem affinity purification (TAP)^16^, and mass spectrometric protein complex^17^ identification have been used for the discovery of PPIs.

However, these experimental methods to detect PPI have some limitations, such as being costly and time-consuming, which restrict them from exploring the entire PPI networks^18–20^. Moreover, the experimental environment and operational processes influence the outcomes of these methods, which result in the occurrences of high false positives (FP) and false negatives (FN). Therefore, the development of robust computational methods in accurately predicting protein–protein interactions is required in conjunction with experimental methods.

To date, many computational methods have been proposed for the prediction of PPIs. Some of them are used to extract new protein information while other methods try to learn the model using extracted features as inputs. The PPI prediction methods are classified into several categories based on the features of proteins used as input information^21^. These are sequence-based, gene co-expression based, protein tertiary structure-based, etc. The autocovariance (AC)^22^ and conjoint triad (CT)^23^ are two widely used sequence-based methods to predict PPI. The protein’s tertiary structure information is also beneficial in predicting PPI. Various experimental techniques are available to determine the protein’s tertiary structure, such as X-ray crystallography and NMR spectroscopy. But, these methods have some limitations, such as being costly and time-consuming. Some computational methods have been proposed to provide the tertiary structure of protein complexes by docking the structure of individual proteins^24–27^. These methods are designed to provide insights into complex structures of proteins, not for predicting PPI. Several attempts have been made to use the protein’s structure information in combination with other characteristics of proteins to determine the PPIs^28,29^.

Deep learning techniques have performed significantly well in many domains, and their usages in the field of computational biology are increasing day by day. Many researchers have used deep learning techniques to predict labels for PPI. Sun et al.^30^ have used a stacked autoencoder classifier to perform the same. The autocovariance and conjoint triad are two protein sequence coding methods used by them to get input representations for the classifier. Du et al.^31^ introduced deep learning classifier where two separate neural networks are used to process the description of each protein in a pair. Gonzalez-Lopez et al.^32^ adopted a deep recurrent neural network to process the input characteristics. The input to this network is achieved by using embedding techniques. Such computational approaches vary in representations of their features and algorithmic processes.

Researchers have collected multi-modal representations of biomedical data with the help of the latest technologies. For example, one form of representation can be the sequence of amino acids, while another can be a 3D structure visualization for a protein. These two modalities for proteins contain distinct information which complement each other. In recent years, deep learning algorithms make it easier to learn useful features from different modalities. Earlier, some researchers have utilized the availability of multi-modal biomedical data in their work. Lovato et al. have used the multimodal approach for protein remote homology detection^33^.

In this work, we have used a multimodal approach that integrates sequential and structural information of proteins to predict PPI. The structural information is retrieved from RCSB Protein Data Bank (PDB; http://www.rcsb.org/pdb/) and is stored in source files with extension .pdb that primarily contain atoms present in protein and their coordinates in 3D space. Various programs are available to visualize the protein structure using the coordinates of atoms stored in a file. For our purpose, we have used the volumetric representation^34^ to visualize the protein’s structure. In volumetric representation, the structure of the object is discretized spatially as binary voxel. If the voxel is occupied, then it is 1 otherwise 0. Hydropathy^35^, isoelectric, and charge are some biological indicators of amino acids. It is believed that these attributes of amino acids play essential roles in determining the interaction between protein sequences^36^. We have also incorporated these attributes into the representation model of proteins and obtained the other three representations of the protein. To extract features from these volumetric representations of proteins, a pre-trained ResNet50 model is utilized. Autocovariance (AC) and conjoint triad (CT) are two popular sequence-based methods to extract features from protein sequences. We have added these features to the input feature set as other modality. So, the input to the model (LSTM based classifier) is the concatenation of features extracted from structural and sequence information of proteins in pairs.

The experimental results show that the proposed method to predict PPI can be used as a complement to the experimental techniques. To train our proposed model, we have used the human PPI dataset, having 25,493 samples. Out of which, 18,025 are positive pairs, and 7468 are negative pairs. Our approach achieves an accuracy of 0.9720, sensitivity of 0.9807, specificity of 0.9504, precision of 0.9799, F-score of 0.9803, and Matthews Correlation Coefficient of 0.9317 on the test set. The proposed framework to predict human PPIs is compared with the method proposed by Sun et al.^30^, which has achieved an accuracy of 0.9683 using autocovariance sequence-based information and 0.9447 using conjoint triad sequence-based information on the test set. To check the proposed approach’s efficacy, we have trained a model on the second PPIs dataset, i.e. *Saccharomyces cerevisiae*. The obtained results are compared with some existing deep-learning-based classifiers^31,37^ trained on the same dataset. The comparison shows that our method outperforms most of the current methods.

## Materials and methodology

In this section, we have discussed how the proposed approach works in predicting protein–protein interactions. This approach is based on multimodal information that integrates sequence-based and 3D structural information of proteins. The working of this model is divided into two phases. In the first phase, we extract features from different modalities of proteins. For structural features, we have converted the coordinates of atoms in protein into several visual representations. Then features are extracted from these representations using a pre-trained ResNet50 model. For sequence-based features, we have used autocovariance and conjoint triad methods. In the second phase, we have utilized these multimodal features by feeding them into deep learning classifiers to predict the correct labels for the PPIs problem.

### Dataset

The Pan’s PPI dataset^19^ consists of positive samples as well as negative samples. The positive pairs belong to the human protein reference database (HPRD, 2007 version). After the removal of duplicate pairs and the protein pairs having odd symbols like *U* and *X*, a total of 36,545 positive protein pairs remains. The negative samples are generated by pairing proteins from different subcellular locations. The information regarding the protein’s subcellular location is obtained from the Swiss-Prot database, version 57.3. After performing some pre-processing on these proteins, such as removal of proteins with multiple subcellular locations or annotated with fragment or having residues length less than 50, a total of 2184 proteins from different subcellular locations are obtained. This pre-processing step also makes sure that all proteins are human proteins. Then a random pairing between proteins from different subcellular locations is done, which is followed by the addition of some negative pairs from^38^. As a result, we have a total of 36,480 negative pairs. The removal of protein pairs having unknown symbols like U and X gives a total of 36,323 negative pairs. Finally, the benchmark dataset consists of 36,545 positive pairs and 36,323 negative pairs.

The second PPI dataset that we have used in this work is the protein pairs of *Saccharomyces cerevisiae*. It can be downloaded from the Database of Interacting Proteins (DIP; version 20160731), which contains 22,975 interacting protein pairs. After removing proteins with less than 50 amino acids followed by cluster analysis of the CD-HIT program^39^, a nonredundant subset with the sequence identity level of 40% is generated with 17,257 positive pairs. The non-interacting pairs are obtained by pairing the proteins from different subcellular localizations. The information about proteins' subcellular localization is available in the Swiss-Prot database. After meeting some requirements such as the non-interacting pairs should not appear in the positive dataset^22^, and the number of protein pairs taken at each subcellular location should not exceed 2500, we have 48,594 negative pairs. The positive and negative protein pairs are combined, which gives a total of 65,851 protein pairs.

There is a limitation to the availability of protein’s tertiary structure information for all the two datasets’ proteins used in this experiment. The structure information is available only for 10,359 protein sequences in Pan’s PPI dataset and for 1308 proteins in the *Saccharomyces cerevisiae* dataset. As a result, we have 25,493 pairs in Pan’s PPI dataset, out of which 18,025 are positive, and 7468 are negative. The *Saccharomyces cerevisiae* dataset has 10,579 protein pairs with 4314 as positive samples and 6265 as negative samples.

### Evaluation criteria

In this experiment, we have used a repeated 3-fold cross-validation (CV) method and a train-test split method to estimate the performance of the model. The 3-fold CV randomly divides the whole dataset into three independent subsets of equal sizes. Each time one subset is used as the test set, and the remaining two subsets are used to train the model. This process is repeated three times so that each subset gets a chance to be the test set once. The 3-fold CV may suffer from the noisy estimation of model’s performance as the results from different splits of data might be very different. To avoid this, we repeat the 3-fold CV method three times, known as repeated 3-fold cross-validation. To get the final results, we take the average and standard deviations of three experiments from all runs. The train-test split divides the dataset into a training set to train the model and test set to measure the model’s performance. Since the PPI problem comes under the category of binary classification problem so the system output must be classified as one of the four types. These are:

- True Positive (TP): When the system accurately categorizes interacting pairs to be interacting.
- False Positive (FP): It is the case where non-interacting pairs are wrongly classified as interacting pairs.
- True Negative (TN): Represents the situation where the system correctly classifies non-interacting pairs to be non-interacting.
- False Negative (FN): If interacting pairs are wrongly categorized as non-interacting.

Accuracy, sensitivity, specificity, precision, F-score, Matthews correlation coefficient (MCC), area under Receiver Operating Characteristic curve (AUROC), and area under Precision-Recall curve (AUPRC) are some widely used evaluation criteria that we have used to measure the performance of the proposed approach. These are defined below:

1 $$\begin{aligned} { Accuracy}= & {} \frac{{ TP} + { TN}}{{ TP} + { FP} + { TN} + { FN}} \end{aligned}$$

2 $$\begin{aligned} { Sensitivity}= & {} \frac{{ TP}}{{ TP} + { FN}} \end{aligned}$$

3 $$\begin{aligned} { Specificity}= & {} \frac{{ TN}}{{ TN} + { FP}} \end{aligned}$$

4 $$\begin{aligned} { Precision}= & {} \frac{{ TP}}{{ TP} + { FP}} \end{aligned}$$

5 $$\begin{aligned} { F-Measure}= & {} \frac{2*{ Precision}*{ Recall}}{{ Precision} + { Recall}} \end{aligned}$$

6 $$\begin{aligned} { MCC}= & {} \frac{{ TP} \times { TN} - { FP} \times { FN}}{\sqrt{({ TP}+{ FP})({ TP}+{ FN})({ TN}+{ FP})({ TN}+{ FN})}} \end{aligned}$$

The accuracy represents the proportion of samples that are correctly classified to the total number of samples. It works well when the datasets are balanced. Sensitivity is the true positive rate. The higher value of sensitivity shows the potential of a classifier to distinguish positive data points. Specificity is the false positive rate. The higher value of specificity represents the ability of a classifier to identify negative data points. F-score quantifies the robustness of the model. The higher the value more robust is the model. MCC calculates the correlation coefficient between the actual class and predicted class. It gives a value between -1 to 1 (1 represents perfect classification and -1 indicates completely wrong classification) and suitable when both classes are of interest. ROC curves and PR curves are the graphical illustrations of the performance of the binary classifier. ROC curve shows the trade-off between TP and FP rates, whereas the PR curve depicts the trade-off between precision and recall of a classifier at different thresholds. The values of the area under these curves are used to compare different classifiers. For imbalanced datasets, PR curves work well, and for balanced datasets, ROC curves are suitable.

### Voxel-based protein structure

**Figure 1:**
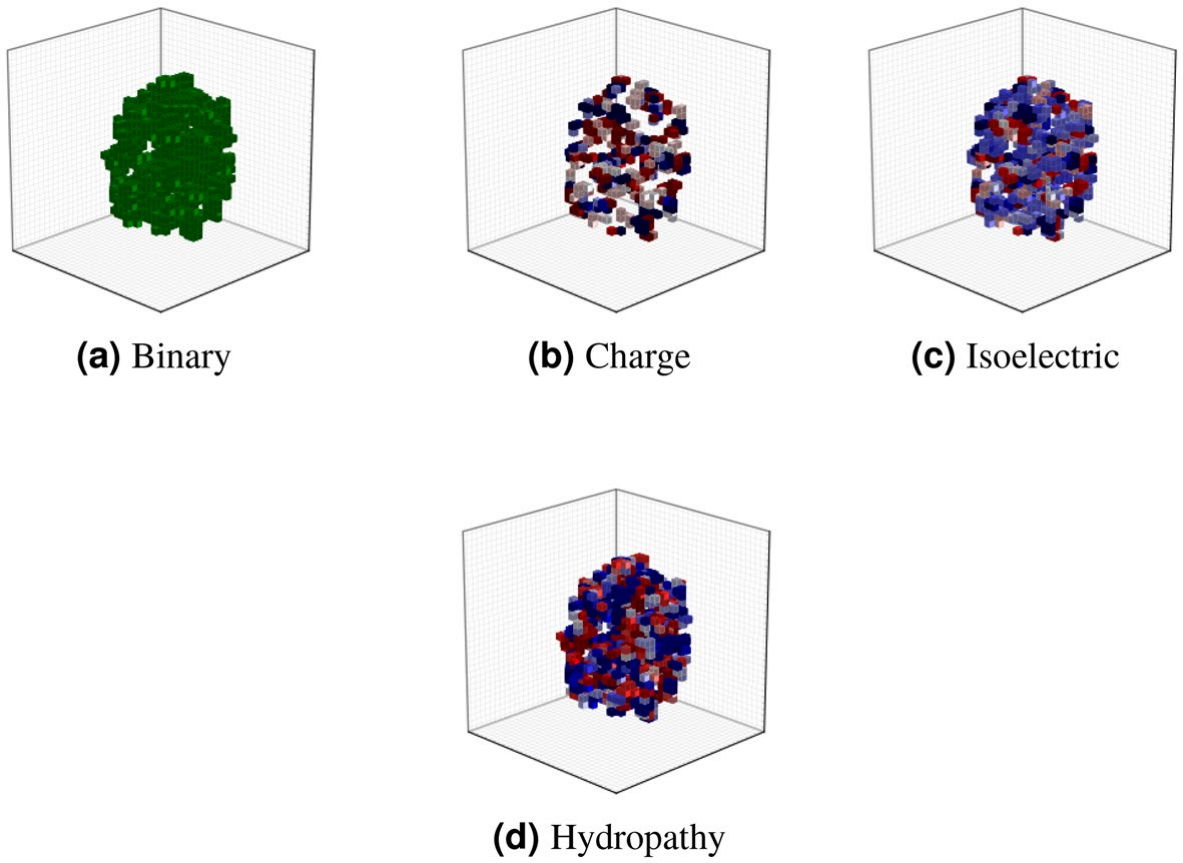
Illustration of binary and several other attributes’ volumetric representation for enzyme 1A00.

The protein’s tertiary structure information is stored in a text file that contains atoms and their (x,y,z) coordinates in space. Each protein is represented as a binary volumetric shape with volume elements such as voxel fitted in a cube *V* of a fixed grid size *l* in the three dimensions. Nearest neighbor interpolation is used to obtain the continuity between voxels, such that for (i, j, k) $\in [0; l-1]^3$, a voxel of vertices

$$\begin{aligned} (i + \delta x, j + \delta y, \delta k + z) | (\delta x, \delta y, \delta z) \in \{0, 1\}^ 3 \end{aligned}$$

takes the value 1 if the backbone of the enzyme passes through the voxel, and 0 otherwise. In this experiment, we have ignored the side chains of protein. We have considered only the backbone atoms such as carbon, nitrogen, and calcium to get the representation of the protein. The binary representation of protein tells only about the shape. Hydropathy index, isoelectric points, and charge are some biological indicators. These indicators describe the local properties of the protein’s building block, i.e., amino acids. These attributes are incorporated into a representation model, which gives us some other useful representations of the protein. So, we have one binary and three attribute volumetric representations for each protein, as depicted in Fig. 1. The various steps involved in getting these volumetric representations of proteins are as follows:

- Extract the 3D coordinates of only the backbone atoms of protein from a text file (.pdb) that contains information about the protein’s tertiary structure. Also, the attribute values for each amino acid of a protein are extracted.
- The coordinates and attribute values obtained from interpolation between consecutive atoms $(A_i, A_{i+1})$ of the backbone are added. These interpolated points are computed as:
  7 $$\begin{aligned} \frac{(p-k+1)*A_i + k*A_{i+1}}{p+1} \end{aligned}$$
  where the value of k varies from 1 to p.
- After this, the centering of coordinates on (0,0,0) is performed.
- Then, the scaling of these coordinates is done by multiplying the coordinates with a value given as:
  8 $$\begin{aligned} \lambda =\left\lfloor \frac{l}{2}-1 \right\rfloor *\frac{1}{R_{max}} \end{aligned}$$
  where l is the grid size and $R_{max}$ is the radius of the sphere that should be fitted into volume V.
- Coordinates are converted into binary voxels and voxels with attributes values.
- Finally, the voxels having no direct neighbor are removed.

In this work, the values chosen for p, $R_{max}$, and l are 5, 40, and 32, respectively^40^.

### Autocovariance

Autocovariance^22^ is a sequence-based method to encode proteins. Among the sequence-based coding scheme, it is one of the widely used processes. It explains the interaction and correlation between variables at different positions in a sequence. The following equation is used to convert the protein sequences into a vector:

9 $$\begin{aligned} AC_{lag,n} = \frac{1}{l-lag}\sum _{m=1}^{l-lag}\left( P_{m,n}-\sum _{m=1}^{l}P_{m,n}\right) *\left( P_{(m+lag),n}-\sum _{m=1}^{l}P_{m,n}\right) \end{aligned}$$

where *P* represents the protein sequence, *l* is the length of the sequence *P*, *m* refers to the location of amino acid in the sequence *P*, *n* is the *n*-th descriptor, $P_{m,n}$ is the normalized *n*-the descriptor value for *m*-th amino acid, and lag refers to the value of the lag. This equation transformed the protein sequences of variable length into vectors of equal size, i.e., $(n \times lag)$. In this study, the value for *n* is taken as 7 as it refers to the seven physicochemical properties of twenty amino acids, and the value chosen for the lag is 30^22^. These values provide a vector with 210 $(7 \times 30)$ elements for each protein sequence.

### Conjoint triad

Conjoint triad^23^ is another popular sequence-based method to convert protein sequences into vectors of numbers. This process of transforming sequences of symbols into vectors of numbers is divided into several steps. First, based on the dipole and side-chain volumes of all twenty amino acids, they are clustered into seven groups. Then, each amino acid of a sequence is replaced by its cluster number. After that, a window of size 3-amino acids is used to slide from N-terminus to C-terminus across the whole sequence. This window slides one step at a time. The total possible combinations with window size 3 and 7 clusters are 343 $(7 \times 7 \times 7)$. So, each protein sequence is represented as a vector with 343 elements. The vector elements represent the count of all combinations across the protein sequence.

### Residual network

A convolutional neural network (CNN), an example of a deep learning model is used to extract features from images. In recent years, various CNN architectures have been available to obtain low/mid/high level features and are widely used in image classification tasks. These architectures come under the category of deep convolutional network. Residual network^41^, also known as ResNet, is the subclass of the deep CNN. In theory, a deeper network means getting better accuracy. But in reality, a deep network may suffer from the problem of vanishing/exploding gradient problem and degradation of training accuracy during the convergence of the neural network. Several methods, like Batch normalization, are used to solve the problem of the vanishing/exploding gradient problem. To address the problem of accuracy degradation, ResNet introduced the concept of skip connection. In a deep convolutional neural network, several layers are stacked which make up the process of learning features during training. But in a residual network, the objective is to learn some residual. Let *H*(*x*) be the mapping of input x obtained by stacking few layers. Then the residual function *F*(*x*) is defined as:

10 $$\begin{aligned} F(x):= H(x) - x \end{aligned}$$

So, *H*(*x*) can be written as $F(x) + x$. Here it is assumed that both *H*(*x*) and *x* have the same dimension.

**Figure 2:**
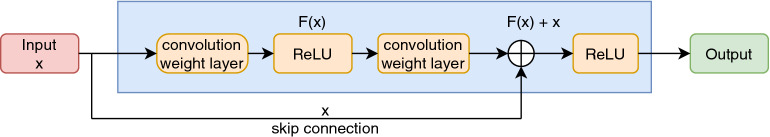
Residual block with two layers.

In a feed-forward neural network, $F(x) + x$ is expressed by using skip connection. Skip connection as the name itself suggests that they skip one or more layers. In the case of ResNet, these connections are used to execute identity mapping. The output of this connection is added to the output of stacked layers, as depicted in Fig. 2. Implementation of skip connection does not involve extra parameters, and computational complexity also remains the same as before. The building block of the residual network is defined as:

11 $$\begin{aligned} y = F(x, {W_i}) + x \end{aligned}$$

where *x* is the input to the layers considered, and *y* represents the output vector. $F(x, {W_i})$ is the residual mapping function that needs to be learned. The residual function *F* is flexible in terms of the number of layers. For two layers, it is described as $F = W_2(\sigma (W_1(x))$, where $W_1$ and $W_2$ are weight matrices, $\sigma$ is the ReLU activation. The operation $F + x$ is achieved by skip connection and element-wise addition. After performing $F + x$ operation, the non-linearity is added by using ReLU activation $(\sigma (F+x))$. For the cases where both *F* and *x* have the same dimension, the Eq. (11) works fine. While in cases where the dimensions differ, we use a modified form of this equation, as shown below:

12 $$\begin{aligned} y = F(x, {W_i}) + W_sx \end{aligned}$$

where $W_s$ is the square matrix used to match the dimensions of *F* and *x*.

In this experiment, we have used the ResNet50 pre-trained model to extract structural features. Here, the number ‘50’ represents the total number of layers it has. The process of feature extraction and concatenation for all four volumetric representations of proteins in pairs are depicted in Fig. 4.

### LSTM network

**Figure 3:**
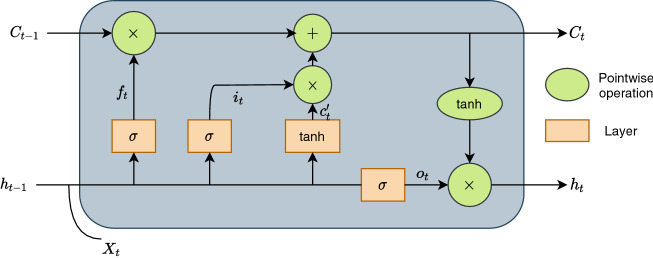
Memory block cell of LSTM network.

LSTM stands for Long Short Term Memory network. It is a type of recurrent neural network (RNN). RNN network suffers from the problem of long-term dependencies. LSTM network is designed to solve this long-term dependency problem of RNN. RNN struggles to remember the information for longer periods, whereas, in the case of LSTM, it is their default behavior. Both RNN and LSTM have the chain of repeating neural network modules, but they differ in their internal structure. The critical components of the LSTM network are the cell state and its several gates, as depicted in Fig. 3. These gates include an input gate, output gate, and forget gate. The cell state is considered as the memory of the network. The job of the forget gate is to decide what information to keep and what to throw away. For that purpose, it takes into consideration the information from the previous hidden state represented as $h_{t-1}$ and the current input $x_t$. These are then passed through a sigmoid function, which gives a number between 0 and 1. A value closer to 0 leads to the removal of the very information, while a value closer to 1 means to keep it. The forget gate is described as:

13 $$\begin{aligned} f_t = \sigma (W_f[h_{t-1}, x_t] + b_f) \end{aligned}$$

where $W_f$ means weight matrix, and $b_f$ is the bias of the forget gate network. The input gate of the LSTM network is used to update its cell state. Like forget gate, it takes previous hidden state information, $h_{t-1}$, and current input, $x_t$, and passes them through sigmoid function. The input gate is defined as:

14 $$\begin{aligned} i_t = \sigma (W_i[h_{t-1}, x_t] +b_i) \end{aligned}$$

where $W_i$ and $b_i$ are the weight matrix and bias vector of the input gate, respectively. The candidate cell state, $c'_t$ with $W_c$ as the weight matrix and $b_c$ as the bias term are defined as:

15 $$\begin{aligned} c'_t = tanh(W_c[h_{t-1}, x_t] +b_c) \end{aligned}$$

So, the actual cell state, $C_t$, at timestamp *t* is defined as:

16 $$\begin{aligned} C_t = f_t \times C_{t-1} + i_t \times C'_t \end{aligned}$$

The output gate is responsible for producing the next hidden state. With $W_o$ as weight matrix and $b_o$ as the bias term, it is described as:

17 $$\begin{aligned} o_t = \sigma (W_o[h_{t-1}, x_t] +b_o) \end{aligned}$$

which gives us the next hidden state, defined below:

18 $$\begin{aligned} h_{t} = o_t \times tanh(C_t) \end{aligned}$$

Here, $\times$ and + represent point-wise multiplication and addition, respectively.

**Figure 4:**
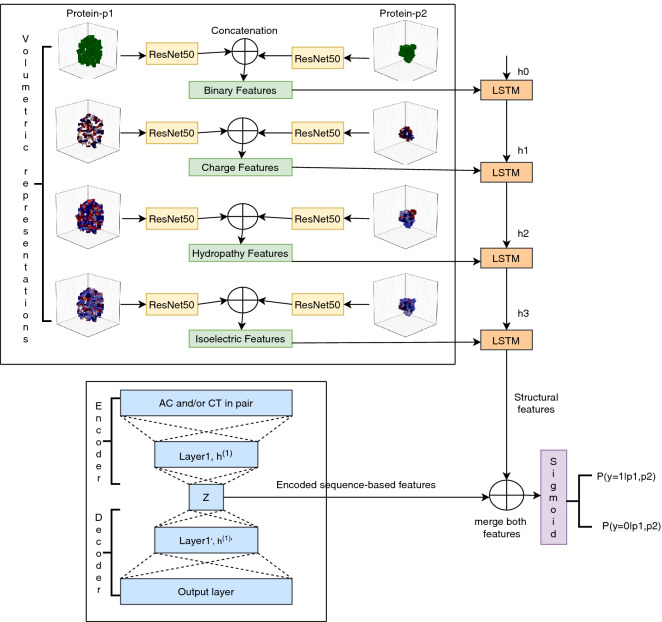
The diagram depicting working of proposed method.

In this experiment, we have used four visual representations for each protein. These are passed through the ResNet50 pre-trained model individually, and a set of feature vectors are generated, each with length 2048. These structural feature vectors of proteins in pairs are concatenated, which gives feature vectors with 4096 elements. Then these four feature vectors are fed into the LSTM layer, which gives a hidden representation of the set of feature vectors at last timestamp. After that, the encoded sequence-based information is concatenated with a hidden representation of structural characteristics. For the encoding purpose, we use a stacked autoencoder having one hidden layer. Finally, these concatenated features are input to a sigmoid layer predicting the output labels for PPI. A value higher than 0.5 means positive class, while less than 0.5 shows negative class. Here, a positive class means that proteins in pairs are interacting with each other. The overall working of the proposed framework to predict PPI is depicted in Fig. 4.

## Results and discussion

This section summarizes the experimental results obtained by the proposed method on the PPIs datasets. We compare the results obtained with those of state-of-the-art deep-learning-based classifiers to illustrate the efficacy of the proposed approach. The models used in this work are implemented in Keras (Python-based framework).

### Prediction performance of proposed model

We have first trained a multi-layer perceptron neural network on each feature set separately of the human PPIs dataset. This neural network consists of an input layer, two hidden layers followed by an output layer. Table 1 summarizes the results of the average of repeated 3-fold cross-validation, with the number of repeats is 3. Since we have multiple feature sets for the PPI task, we have considered the average of these feature sets and trained the neural network on that average feature set. The results are mentioned in Table 1. The obtained results show that the binary 3D structural information, when combined with amino acids’ local properties, gives better results than the binary structural information. The neural network trained on charge-based features provides the highest value for MCC’s average, i.e., 0.8577. The average MCC values for other models are 0.7658, 0.8462, 0.8471, and 0.7843, respectively. MCC value is beneficial to compare different models when both classes of a binary classifier are of equal importance. We have also calculated other performance measures such as F-score to measure the model’s stability, specificity to check its prediction ability in case of negative samples, sensitivity, precision, area under the ROC curve, and area under the PR curve. The area under the PR curve is suitable for an imbalanced dataset. It can be seen from Table 1 that all models are relatively good at predicting positive samples (sensitivity) than predicting negative samples (specificity). The average sensitivity and specificity values of different MLP-based models are {0.9511, 0.9538, 0.9565, 0.9646, 0.9122} and {0.7862, 0.9071, 0.8827, 0.8672, 0.8869}, respectively.

**Table 1:** The average repeated 3-fold cross-validation results on different features of proteins using multi-layer perceptron.

**Features**	**Accuracy**	**Sensitivity**	**Specificity**	**Precision**	**F-score**	**MCC**	**AUROC**	**AUPRC**
Binary	0.9027	0.9511	0.7862	0.9187	0.9342	0.7658	0.9676	0.9836
Charge	**0.9400**	0.9538	**0.9071**	**0.9617**	**0.9574**	**0.8577**	0.9761	0.9875
Isoelectric	0.9349	0.9565	0.8827	0.9527	0.9539	0.8462	0.9776	0.9881
Hydropathy	0.9360	**0.9646**	0.8672	0.9473	0.9552	0.8471	**0.9787**	**0.9887**
Avg of all Features	0.9048	0.9122	0.8869	0.9528	0.9308	0.7843	0.9627	0.9810

The best results are marked in bold.

#### Experimental results on Pan’s PPIs dataset

From Table 1, we can see that the multi-layer perceptron neural network taking the average of feature sets as an input did not achieve good results. Also, previous studies suggest that if we integrate features obtained from different modalities and then utilize these combined features to predict PPI may give better results. Motivated by this, we have used autocovariance and conjoint triad methods for coding protein sequences and used them as additional features sets. We also need to capture better representations for structural feature sets. To achieve our goal, we have implemented LSTM based classifier. It takes a different feature set at different timestamps. Here, the value for timestamps is four, as we have four feature sets (binary, hydropathy-based, isoelectric-based, and charge-based). The hidden state value at last timestamp is then concatenated with encoded AC and CT features. All the feature sets should have the same dimension when concatenated along the axis of the number of features extracted by different methods. For that purpose, an autoencoder is used to encode the features obtained by AC and CT. Finally, these concatenated features, consisting of structural and sequence-based information, are fed into the sigmoid layer (output layer) to predict PPIs. Tables 2, 3, 4 summarize the repeated 3-fold cross-validation results achieved by different combinations of the concatenation of feature sets (bimodal) on the human PPIs dataset. Unlike multi-layer perceptron classifiers trained on different feature sets individually, the prediction ability of the proposed framework is significantly well in both cases (positive samples and negative samples). The average values of sensitivity and specificity achieved by the combinations {structural+AC, structural+CT, structural+AC+CT} are {0.9768, 0.9742, 0.9784} and {0.9486, 0.9632, 0.9588}, respectively. The average accuracy, F-score and Matthews Correlation Coefficient (MCC) of these combinations are {0.9686, 0.9720, 0.9726}, {0.9777, 0.9793, 0.9806} and {0.9243, 0.9309, 0.9343}, respectively.

**Table 2:** The repeated 3-fold cross-validation results on Human PPI dataset using LSTM-based classifier that integrates structural features and autocovariance.

**#Repeat**	**Test set**	**Accuracy**	**Sensitivity**	**Specificity**	**Precision**	**F-score**	**MCC**	**AUROC**	**AUPRC**
Repeat 1	1	0.9600	0.9679	0.9409	0.9753	0.9716	0.9040	0.9803	0.9866
	2	0.9738	0.9830	0.9514	0.9799	0.9815	0.9365	0.9904	0.9949
	3	0.9771	0.9829	0.9630	0.9847	0.9838	0.9447	0.9916	0.9949
Repeat 2	1	0.9522	0.9582	0.9377	0.9738	0.9659	0.8863	0.9773	0.9852
	2	0.9658	0.9694	0.9570	0.9820	0.9756	0.9183	0.9861	0.9907
	3	0.9781	0.9815	0.9699	0.9874	0.9845	0.9474	0.9925	0.9958
Repeat 3	1	0.9536	0.9770	0.8971	0.9582	0.9675	0.8870	0.9778	0.9857
	2	0.9765	0.9890	0.9462	0.9779	0.9834	0.9429	0.9919	0.9956
	3	0.9799	0.9824	0.9739	0.9891	0.9857	0.9517	0.9946	0.9970
Mean		**0.9686**	**0.9768**	**0.9486**	**0.9787**	**0.9777**	**0.9243**	**0.9869**	**0.9918**
Std. deviation		0.0103	0.0092	0.0216	0.0087	0.0073	0.0246	0.0064	0.0045

The average results are marked in bold.

**Table 3:** The repeated 3-fold cross-validation results on Human PPI dataset using LSTM-based classifier that integrates structural features and conjoint triad.

**#Repeat**	**Test set**	**Accuracy**	**Sensitivity**	**Specificity**	**Precision**	**F-score**	**MCC**	**AUROC**	**AUPRC**
Repeat 1	1	0.9629	0.9645	0.9590	0.9827	0.9735	0.9121	0.9847	0.9892
	2	0.9727	0.9762	0.9643	0.9851	0.9806	0.9346	0.9934	0.9965
	3	0.9774	0.9808	0.9691	0.9871	0.9840	0.9457	0.9952	0.9977
Repeat 2	1	0.9598	0.9512	0.9498	0.9786	0.9647	0.8845	0.9801	0.9873
	2	0.9760	0.9840	0.9566	0.9821	0.9830	0.9420	0.9906	0.9941
	3	0.9807	0.9827	0.9759	0.9900	0.9863	0.9537	0.9960	0.9980
Repeat 3	1	0.9648	0.9675	0.9582	0.9824	0.9749	0.9163	0.9852	0.9911
	2	0.9712	0.9735	0.9654	0.9855	0.9795	0.9312	0.9915	0.9949
	3	0.9825	0.9874	0.9707	0.9878	0.9876	0.9577	0.9968	0.9982
Mean		**0.9720**	**0.9742**	**0.9632**	**0.9846**	**0.9793**	**0.9309**	**0.9904**	**0.9941**
Std. deviation		0.0075	0.0108	0.0076	0.0033	0.0068	0.0218	0.0055	0.0038

The average results are marked in bold.

**Table 4:** The repeated 3-fold cross-validation results on Human PPI dataset using LSTM-based classifier that integrates structural features with autocovariance and conjoint triad.

**#Repeat**	**Test set**	**Accuracy**	**Sensitivity**	**Specificity**	**Precision**	**F-score**	**MCC**	**AUROC**	**AUPRC**
Repeat 1	1	0.9606	0.9584	0.9658	0.9854	0.9717	0.9076	0.9847	0.9904
	2	0.9760	0.9810	0.9639	0.9850	0.9830	0.9422	0.9940	0.9968
	3	0.9809	0.9865	0.9675	0.9865	0.9865	0.9540	0.9949	0.9972
Repeat 2	1	0.9648	0.9814	0.9249	0.9693	0.9753	0.9145	0.9834	0.9905
	2	0.9728	0.9787	0.9586	0.9828	0.9807	0.9346	0.9901	0.9944
	3	0.9815	0.9870	0.9683	0.9869	0.9869	0.9554	0.9957	0.9978
Repeat 3	1	0.9590	0.9695	0.9337	0.9725	0.9710	0.9014	0.9803	0.9863
	2	0.9748	0.9742	0.9763	0.9900	0.9820	0.9402	0.9917	0.9950
	3	0.9831	0.9885	0.9699	0.9875	0.9880	0.9591	0.9967	0.9984
Mean		**0.9726**	**0.9784**	**0.9588**	**0.9829**	**0.9806**	**0.9343**	**0.9902**	**0.9941**
Std. deviation		0.0086	0.0091	0.0164	0.0067	0.0061	0.0203	0.0056	0.0039

The average results are marked in bold.

Table 5 presents the results obtained on the test set of the human PPIs dataset for different bimodal feature combinations. We randomly select 80% of the dataset as the training set. The remaining 20% is used as a test set to check the trained model’s predictive capability on unseen data. The results of the models trained on different bimodal feature combinations are comparable. The accuracy, F-score, Matthews Correlation Coefficient (MCC), area under the ROC curve (AUROC), and area under the PR curve (AUPRC) of these models are {0.9692, 0.9706, 0.9720}, {0.9785, 0.9794, 0.9803}, {0.9246, 0.9282, 0.9316}, {0.9831, 0.9831, 0.9839}, and {0.9897, 0.9886, 0.9887}, respectively.

**Table 5:** The prediction performances on test set of Human PPI dataset for different multimodal feature combinations.

**Modal**	**Accuracy**	**Sensitivity**	**Specificity**	**Precision**	**F-score**	**MCC**	**AUROC**	**AUPRC**
Structural+AC	0.9692	**0.9829**	0.9354	0.9741	0.9785	0.9246	0.9831	**0.9897**
Structural+CT	0.9706	0.9804	0.9463	0.9783	0.9794	0.9282	0.9831	0.9886
Structural+AC+CT	**0.9720**	0.9807	**0.9504**	**0.9799**	**0.9803**	**0.9316**	**0.9839**	0.9887

The best results are marked in bold.

#### Experimental results on *Saccharomyces cerevisiae* PPIs dataset

The *Saccharomyces cerevisiae* dataset has 4314 interacting protein pairs and 6265 non-interacting protein pairs. Since we have less number of positive samples, we randomly select 1951 positive samples from the dataset. We mix these randomly selected positive pairs to the dataset so that the final dataset has a 1:1 ratio of positive samples and negative samples. Then, we randomly split the final dataset having 12,530 samples into two parts (80% and 20%). The first part, i.e., 80% of the final dataset, is used to train the model. The remaining 20% is used as a test set to analyze the performance of the trained model. Table 6 presents the results of the proposed approach on the test set for each bimodal feature combinations. The accuracy, F-score, and Matthews Correlation Coefficient (MCC) for each model trained on different feature combinations are {0.9206, 0.9266, 0.9370}, {0.9177, 0.9263, 0.9359}, and {0.8424, 0.8532, 0.8740}, respectively.

**Table 6:** The prediction performances on test set of *Saccharomyces cerevisiae* PPI dataset for different multimodal feature combinations.

**Modal**	**Accuracy**	**Sensitivity**	**Specificity**	**Precision**	**F-score**	**MCC**	**AUROC**	**AUPRC**
Structural+AC	0.9206	0.8922	**0.9485**	**0.9446**	0.9177	0.8424	0.9764	0.9776
Structural+CT	0.9266	**0.9300**	0.9232	0.9226	0.9263	0.8532	0.9777	0.9796
Structural+AC+CT	**0.9370**	0.9276	0.9462	0.9443	**0.9359**	**0.8740**	**0.9781**	**0.9800**

The best results are marked in bold.

### Results with varying modalities

**Figure 5:**
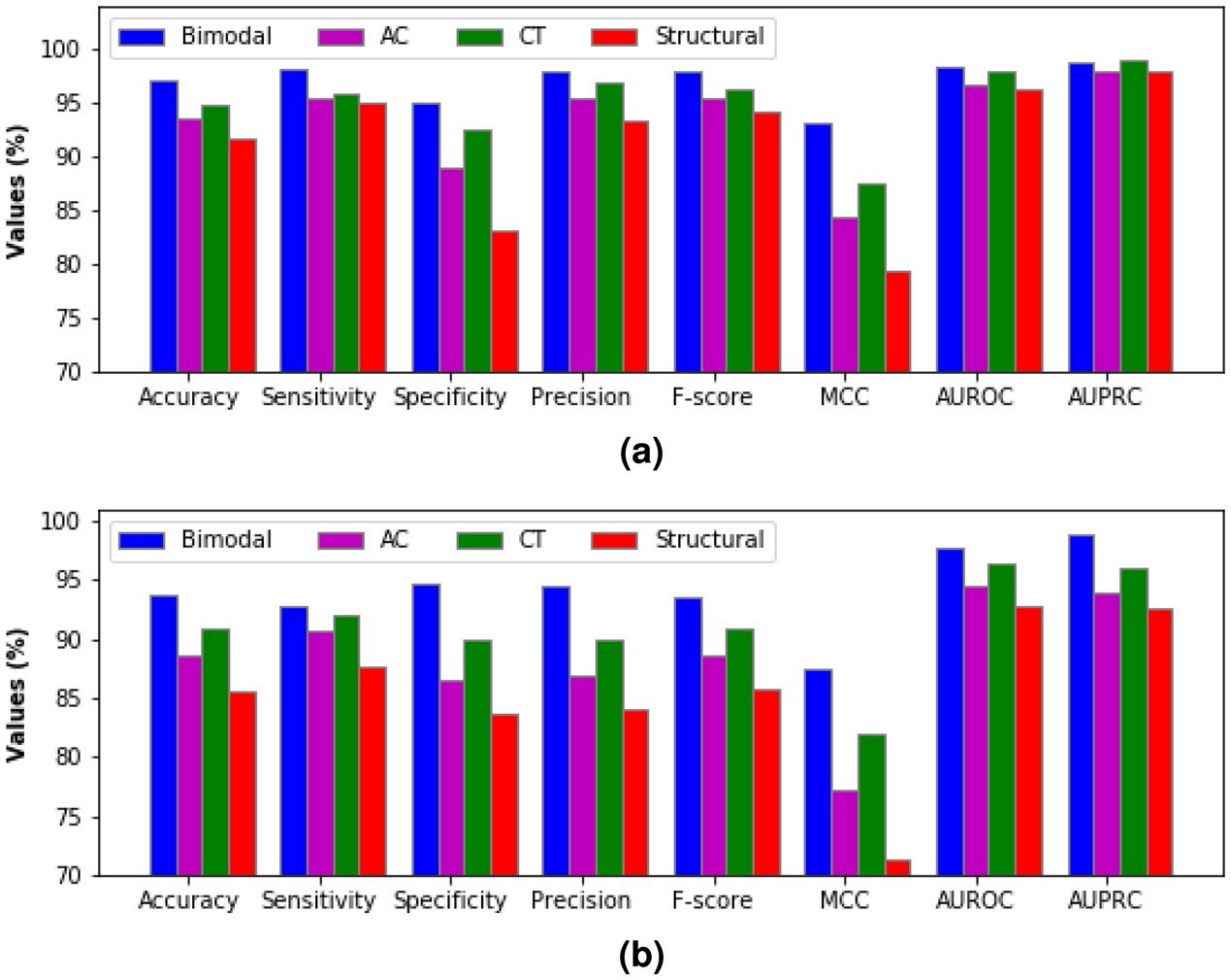
Illustration of the performance of models trained on different feature combinations. (**a**) Human PPIs dataset, (**b**) *Saccharomyces cerevisiae* PPIs dataset.

Tables 7 and 8 summarize the results for a unimodal and bimodal feature sets on test data of the human PPIs dataset and *Saccharomyces cerevisiae* PPIs dataset, respectively. The term unimodal means we have used only one mode of information, either sequence-based or structural features, to train the proposed model. The term bimodal means that we have utilized two types of information representing proteins to get the final feature vectors used as input to the model. For encoded features obtained from sequence-based methods (AC and CT), we have used Sun et al.^30^ approach to get the values of performance metrics on the test sets of these two datasets. For unimodal structural features, the hidden state representation at the last timestamp, as shown in Fig. 4, is fed directly into the sigmoid layer (output layer). The results show an improvement in classifiers’ predictive potential when both structural and sequence-based features are combined. The values of accuracy, F-score, and MCC of bimodal features {Structural + AC + CT} are 2.43%, 1.71%, and 6.01% higher than unimodal features {CT}, respectively on the test set of human PPIs dataset. The values of accuracy, F-score, and MCC of bimodal features {Structural + AC + CT} are 2.95%, 2.80%, and 6.28% higher than unimodal features {CT}, respectively on the test set of *Saccharomyces cerevisiae* PPIs dataset. Figure 5 depicts the results for different feature combinations in the form of a histogram.

**Table 7:** The results of Human PPIs dataset for different feature combinations on test set.

**Modal**	**Feature combinations**	**Accuracy**	**Sensitivity**	**Specificity**	**Precision**	**F-score**	**MCC**	**AUROC**	**AUPRC**
Unimodal	AC	0.9348	0.9534	0.8907	0.9537	0.9536	0.8440	0.9679	0.9800
	CT	0.9484	0.9578	0.9252	0.9693	0.9635	0.8756	0.9804	**0.9902**
	Structural	0.9167	0.9512	0.8314	0.9330	0.9420	0.7945	0.9619	0.9793
Bimodal	Structural+AC	0.9692	**0.9829**	0.9354	0.9741	0.9785	0.9246	0.9831	0.9897
	Structural+CT	0.9706	0.9804	0.9463	0.9783	0.9794	0.9282	0.9831	0.9886
	Structural+AC+CT	**0.9720**	0.9807	**0.9504**	**0.9799**	**0.9803**	**0.9316**	**0.9839**	0.9887

The best results are marked in bold.

**Table 8:** The results of *Saccharomyces cerevisiae* PPIs dataset for different feature combinations on test set.

**Modal**	**Feature combinations**	**Accuracy**	**Sensitivity**	**Specificity**	**Precision**	**F-score**	**MCC**	**AUROC**	**AUPRC**
Unimodal	AC	0.8855	0.9067	0.8646	0.8683	0.8871	0.7718	0.9447	0.9386
	CT	0.9094	0.9204	0.8987	0.8994	0.9097	0.8191	0.9633	0.9605
	Structural	0.8559	0.8761	0.8361	0.8403	0.8578	0.7126	0.9281	0.9270
Bimodal	Structural+AC	0.9206	0.8922	**0.9485**	**0.9446**	0.9177	0.8424	0.9764	0.9776
	Structural+CT	0.9266	**0.9300**	0.9232	0.9226	0.9263	0.8532	0.9777	0.9796
	Structural+AC+CT	**0.9370**	0.9276	0.9462	0.9443	**0.9359**	**0.8740**	**0.9781**	**0.9880**

The best results are marked in bold.

### Comparison with existing methods

To further evaluate the proposed method’s performance, we have compared our results with those of state-of-the-art deep-learning-based methods^30,31,37^. Table 9 presents the comparison of results between the proposed approach and stacked autoencoder (SAE) based classifier^30^ on the test set of Pan’s human PPIs dataset. The original human PPIs dataset has 72,868 samples, and due to the unavailability of structural information of proteins, we have only 25,493 protein pairs. The test set used by Sun et al. contains 7000 samples (3493 positive pairs and 3507 negative pairs). Our test set contains 5099 samples (3628 positive and 1471 negative). We compare our results with the actual results mentioned in^30^. $SAE\_AC$ is a stacked autoencoder taking inputs extracted using the autocovariance method. $SAE\_CT$ is the stacked autoencoder model whose input is obtained by the conjoint triad method from protein sequences. It can be seen from Table 9 that the proposed approach outperforms the existing method. The accuracy values obtained by the state-of-the-art methods and the proposed method are 0.9682, 0.9447, and 0.9720, respectively. To make the comparison between models fair, we have also trained the state-of-the-art models ($SAE\_AC$ and $SAE\_CT$) on the training set of our dataset. The obtained results on our test set are mentioned in Table 7 (row 1 and row 2). The values of accuracy, sensitivity, specificity, precision, F-score, MCC, and area under the PR curve obtained by the proposed approach are 3.83%, 2.78%, 6.28%, 2.67%, 2.72%, 9.40%, and 0.88% higher than those obtained by $(SAE\_AC)$, respectively.

**Table 9:** Performance comparison between proposed approach and existing methods on the test set of Human PPIs dataset.

**Model**	**Accuracy**	**Sensitivity**	**Specificity**	**Precision**	**F-score**	**MCC**	**AUROC**	**AUPRC**
SAE_AC^30^	0.9682	NA	NA	NA	NA	NA	NA	NA
SAE_CT^30^	0.9447	NA	NA	NA	NA	NA	NA	NA
Proposed approach	**0.9720**	**0.9807**	**0.9504**	**0.9799**	**0.9803**	**0.9316**	**0.9839**	**0.9887**

The best results are marked in bold.

Note: NA means not available.

Table 10 presents the comparison of results between the proposed approach and existing deep-learning-based methods^31,37^ on *Saccharomyces cerevisiae* PPIs dataset. The original dataset contains 65,851 samples with 17,257 positive samples and 48,594 negative samples. After removing the protein pairs for which no structural information is available for any protein in pairs, only 10,579 samples (4314 positive samples and 6265 negative samples) remain. The number of samples in our dataset (10,579 samples) is significantly less than the original dataset (65,851 samples). To prepare our final dataset, we randomly select 1951 positive samples and mix them into the dataset with 10,579 samples. As a result, our final dataset consists of 12,530 samples with a 1:1 ratio of positive and negative samples. DeepPPI-Sep and DeepPPI-Con are two models proposed by Du et al. that follow different architectures. EnsDNN is proposed by Zhang et al., and EnsDNN-Sep and EnsDNN-Con are the two variations of EnsDNN. The results reported in Table 10 are the average of 5-fold cross-validation. The results of the proposed approach are compared against the actual results of Du’s work^31^ and Zhang’s work^37^. The comparison of results shows that our method to predict PPIs outperforms the existing sequence-based methods. The accuracy, area under the ROC curve (AUROC), and MCC of the DeepPPI-Sep, EnsDNN, and the proposed approach are {0.9250, 0.9529, 0.9604}, {0.9743, 0.9700, 0.9904}, and {0.8508, 0.9059, 0.9209}, respectively. From these results, it can be observed that multimodal information of proteins is beneficial in predicting protein–protein interactions.

**Table 10:** Performance comparison between proposed approach and existing methods on *Saccharomyces cerevisiae* PPIs dataset.

**Model**	**Accuracy**	**Sensitivity**	**Specificity**	**Precision**	**F-score**	**MCC**	**AUROC**	**AUPRC**
DeepPPI-Sep^31^	0.9250	0.9056	0.9449	0.9438	NA	0.8508	0.9743	NA
DeepPPI-Con^31^	0.9001	0.8847	0.9160	0.9150	NA	0.8008	0.9576	NA
EnsDNN-Con^37^	0.9068	0.9014	0.9119	0.9119	0.9062	0.8143	0.9645	NA
EnsDNN-Sep^37^	0.9119	0.9223	0.9017	0.9041	0.9129	0.8244	0.9659	NA
EnsDNN^37^	0.9529	0.9512	0.9548	0.9545	0.9529	0.9059	0.9700	NA
Proposed approach	**0.9604**	**0.9634**	**0.9574**	**0.9578**	**0.9606**	**0.9209**	**0.9904**	**0.9909**

The best results are marked in bold.

Note: NA means not available.

### Statistical significance test

The statistical significance test is used to compare different models statistically. We have performed this test on the results obtained by the proposed approach to illustrate that improvements in performance are statistically significant. To accomplish this, we have conducted the experiments 10 times using 3-fold cross-validation. Welch’s *t*-test^42^ with 5% (0.05) significance level is conducted to illustrate that the accuracy values obtained by the proposed approach are not happened by chance. The *t*-test gives *p*-value, which is the probability of the improvements in results just occurred by chance. It the p-value is less than 0.05, it means that the results are statistically significant (rejection of the null hypothesis). A null hypothesis states that there is no significant difference between the results achieved by two different algorithms. Table 11 presents the p-values for different bimodal combinations of the input feature set. All the values mentioned in Table 11 are less than 0.05, indicating the results achieved by the proposed method to predict PPIs are statistically significant.

**Table 11:** *p*-values obtained for different bimodal feature set combinations of two datasets.

**Bimodal combinations**	**Human PPIs**	*** Saccharomyces cerevisiae***
Structural+AC	1.10954e-22	0.0345
Structural+CT	2.31427e-23	2.19183e-06
Structural+AC+CT	2.72237e-24	2.66818e-15

## Conclusion

The study of protein–protein interaction is essential as the various activities and functions of a protein depend on the protein(s) that interact with it. There are various methods available to detect PPIs. But still, there is a scope to improve the prediction capability and robustness of these methods by using multimodal biomedical data and the latest techniques of deep learning. In this work, we have combined different modalities of proteins to improve the prediction capability of the classifier. These modalities include the sequence-based information and structural view of proteins. Deep learning algorithms (ResNet50 and Stacked autoencoder) are used to extract features from these modalities. These features are then used as input to the classifier. The improvements in results attained by our proposed method are statistically significant. The proposed method achieves an average accuracy of 0.9726 of repeated 3-fold cross-validation on the human PPIs dataset with 25,493 samples. Our proposed approach is also compared with some widely used deep-learning-based classifiers that utilize sequence-based information to train the model. The obtained results demonstrate that the proposed approach generally outperforms the existing methods. The significant observation from this study is that the proposed approach can learn useful features from multimodal information of proteins and perform well despite the model being trained on a lesser number of samples. In the future, we will try using some other type of information about proteins and deep learning techniques with the hope of getting better result.
